# The Life Goals Self-Management Mobile App for Bipolar Disorder: Consumer Feasibility, Usability, and Acceptability Study

**DOI:** 10.2196/32450

**Published:** 2021-12-13

**Authors:** Kelly A Ryan, Shawna N Smith, Anastasia K Yocum, Isabel Carley, Celeste Liebrecht, Bethany Navis, Erica Vest, Holli Bertram, Melvin G McInnis, Amy M Kilbourne

**Affiliations:** 1 Department of Psychiatry University of Michigan Ann Arbor, MI United States; 2 Department of Health Management and Policy School of Public Health University of Michigan Ann Arbor, MI United States; 3 VA Ann Arbor Healthcare System United States Department of Veterans Affairs Ann Arbor, MI United States; 4 Department of Learning Health Sciences University of Michigan Ann Arbor, MI United States

**Keywords:** self-management, app, bipolar disorder, symptom management, mental health, feasibility, usability, acceptability, intervention, bipolar, coping, survey, engagement

## Abstract

**Background:**

Life Goals is an evidence-based self-management intervention that assists individuals with bipolar disorder (BD) by aligning BD symptom coping strategies with their personal goals. The intervention can be availed via in-person and telephonic sessions, and it has been recently developed as an individualized, customizable mobile app.

**Objective:**

We examined the feasibility, usability, and acceptability of the Life Goals self-management app among individuals diagnosed with BD who used the app for up to 6 months.

**Methods:**

A total of 28 individuals with BD used the Life Goals app on their personal smartphone for 6 months. They completed key clinical outcome measurements of functioning, disability, and psychiatric symptoms at baseline, 3 months, and 6 months, in addition to a poststudy survey about usability and satisfaction.

**Results:**

Participants used the app for a median of 25 times (IQR 13-65.75), and for a longer time during the first 3 months of the study. The modules on depression and anxiety were the most frequently used, accounting for 35% and 22% of total usage, respectively. Overall, the study participants found the app useful (15/25, 60%) and easy to use (18/25, 72%), and they reported that the screen displayed the material adequately (22/25, 88%). However, less than half of the participants found the app helpful in managing their health (10/25, 40%) or in making progress on their wellness goals (9/25, 36%). Clinical outcomes showed a trend for improvements in mental and physical health and mania-related well-being.

**Conclusions:**

The Life Goals app showed feasibility of use among individuals with BD. Higher user engagement was observed in the initial 3 months with users interested more frequently in the mood modules than other wellness modules. Participants reported acceptability with the ease of app use and satisfaction with the app user interface, but the app showed low success in encouraging self-management within this small sample. The Life Goals app is a mobile health technology that can provide individuals with serious mental illness with more flexible access to evidence-based treatments.

## Introduction

Bipolar disorder (BD), a serious mental illness, affects at least 2% of the general population. It is the sixth leading cause of disability worldwide affecting the 18- to 44-year-old age group [1], a major risk factor for suicide, and the single most expensive mental health condition [2]. As BD is a chronic condition, individuals with BD often require continuous, long-term monitoring and care. However, 40% to 50% of those with any serious mental illness, including BD, do not receive any mental health treatment [3,4] despite the knowledge that evidenced-based interventions improve engagement with providers [5]. Commonly reported barriers to obtaining adequate care include lack of access, cost, and stigma [6-9]. The vast majority of patients with BD receive care in low-resource settings, which include primary care and community mental health clinics [1,2]. These providers are overburdened and lack the capacity to provide consistent monitoring shown to be effective in the management of individuals with BD [3,10-12]. Mobile health (mHealth) technologies have the potential to overcome these barriers and improve access to care [13]. There are many obstacles to implementing mobile and digital health strategies [14]. Therefore, it is imperative that the field adopts digital health strategies that are grounded in evidenced-based research; offers greater flexibility in terms of treatment options; and accommodates long-term use by heterogeneous patient populations, including varying levels of user engagement or commitment.

Existing or widely available mHealth apps for BD that are readily downloadable on smartphones typically only offer mood tracking or symptom monitoring, do not cover other important areas for individuals with BD (eg, medication adherence, sleep, and intervention), or are not supported by empirical evidence. These include apps such as eMoods Bipolar Mood Tracker, Moodlog, UP! – Depression, Bipolar & Borderline Management, and Bipolar Track. Findings of a systematic review of available apps for BD indicate that the most widely available apps do not reference clinical practice guidelines, standard psychoeducation information, or established self-management tools [15]. Other work on existing BD-related apps found that BD consumer needs were not adequately addressed by the currently available apps [16]. The top features included were mood tracking and journaling [17], and it did not provide adequate educational or other intervention content. Of the 100 top-returned publicly available apps for BD that were reviewed, only 56% mention BD in the content or description, and only 1 app was supported by peer-reviewed research. These findings suggest that mHealth tools for BD have not been widely translated into evidence-based, clinically relevant apps that can be made available to the public.

One specific mHealth tool that is empirically supported—the MONARCA app—uses sensors and self-assessments to gather information about the user’s sleep, social activity, and mood, with the goal of providing information to the patients and their providers, but it does not include self-management and educational components [18]. Platforms and databases have been developed to help professionals and consumers access mHealth apps that translate evidenced-based programs and provide guidelines for consumers and help to navigate the mental health app marketplace, including some of the apps listed above [19,20]. However, very few scientifically reviewed apps specifically focus on the needs of individuals with BD, especially beyond mood tracking. One newly developed smartphone-based, self-management intervention for BD (LiveWell) was designed considering empirically supported therapy and included user input in its development, which addresses the need for empirically supported apps to provide self-management components [21]. Therefore, there is a need to focus on the development of other apps to provide options for patients with BD who would like self-management strategies and education, including support with sleep, understanding early warning signs, triggers, and maintaining healthy lifestyles [22,23].

Life Goals Collaborative Care (LGCC) [24] is one such evidence-based intervention based on the chronic collaborative care model that provides proactive care for patients through several components, including patient self-management education; care coordination across providers, predominantly through care manager contacts and improved information systems; and decision support tools for providers. LGCC’s central patient-centered tenet focuses on empowering patient-self management skills through the Life Goals program, which is a series of 6 or more self-management sessions customized to individual needs and focused on mental and physical wellness, understanding symptoms, and setting personal goals. Although a component of LGCC, the Life Goals self-management program can serve as a stand-alone, manualized program for individuals with BD [25], and it has been offered via a user-friendly, provider-facing website that guides the Life Goals provider and their patient to customize an LGCC program and a consumer self-directed guidebook [26].

Self-management programs such as those included within the LGCC have been shown to improve medical and psychiatric outcomes for persons with serious mental illness after 6 months [27,28]. Several randomized trials found that LGCC reduced overall affective symptoms and improved overall role function, quality of life, participant satisfaction, and medical outcomes, compared to usual care [24,27].

Self-management programs are patient-centered and encourage regular engagement with the provider. This is challenging and burdensome for providers to implement in low-resource communities. Community-based practices lack the staffing time and capacity (eg, physical space) to offer self-management sessions such as the original LGCC program. They may also face barriers to reimbursement [29]. At the patient level, access to effective, evidence-based self-management programs are also limited outside of in-person care, which may be challenging to access owing to cost, workforce, and stigma. There is a need to develop self-management programs into mHealth technologies, with the hope that such apps will fill this gap by alleviating the burden on the providers and empower those with serious mental illness by providing them with more flexible access to evidence-based treatments.

Barriers regarding access to care and available mHealth platforms were the impetus for the development of a standalone Life Goals self-management mobile app for persons with BD that can be accessed either in a *direct-to-consumer* format or as an augmentation to *in-person* care. The aim of this study is to evaluate the feasibility, usability, and acceptability of the Life Goals self-management app among individuals diagnosed with BD over a 6-month period. Outcome patterns of key clinical outcomes relating to functioning, disability, and psychiatric symptoms will also be assessed. The Life Goals app adds value to the existing mHealth apps for BD given its evidenced-based content, intervention effectiveness, and functionality of being customizable to the user’s need.

## Methods

### Participant Recruitment

For this pre-post-test study, participants were recruited from the Heinz C. Prechter Longitudinal Study of Bipolar Disorder, an observational, naturalistic cohort study gathering phenotypic and biological data, at the University of Michigan [30], or through the UMHealthResearch web-based tool [31]. Individuals were included in the study if they were aged 18 years or older, had any BD diagnosis (ie, Bipolar I, II, or not otherwise specified [NOS], according to the Diagnostic Statistic Manual version IV [DSM-IV] criteria), were community dwelling (ie, not living in a nursing home or other institution), and were current owners and users of a smartphone. Diagnosis was confirmed using medical records or a diagnostic interview using the Diagnostic Interview for Genetics Study [32] or the Mini-International Neuropsychiatric Interview (MINI) [33]. Exclusion criteria were any serious illness precluding participation in Life Goals components as indicated by the provider, or inability to provide informed consent. One participant was excluded because she did not meet the criteria for BD after completing the MINI. Three participants discontinued from the study before completion owing to time constraints (n=2) or because they had difficulty using the app on their phone (n=1). This study was approved by the University of Michigan Internal Review Board, and all participants provided signed informed consent.

### Smartphone App

The Life Goals app was custom built to use either Apple or Android operating system. Participants were sent invitations to download the app from either Apple App Store or Google Play. Once they logged into the app, they could review the privacy policy (created by University of Michigan’s Office of Technology Transfer), which was also saved under the main menu tab for easy access. Participant data were stored securely on University of Michigan’s approved servers and protected by the university’s security systems. A trained research associate provided training on how to use the app and provided a user manual for troubleshooting. The study team was available to answer questions via phone every business day.

The Life Goals app includes succinct, 5- to 10-minute-long modules that provide self-management activities for managing everyday needs of individuals with BD, including mood symptom coping strategies, stigma concerns, emotional self-awareness and family support, anger and irritability, and preparation for doctor’s visits. The content and wording were adapted from the original LGCC program. We encouraged participants to engage with Life Goals modules once a day, but they were ultimately self-guided, interactive modules completed at the user’s own pace. Participants could type in free-text personal responses for questions asked by the app. All participants were required to complete the introduction module first, where they learned about self-management, collaborative care, stigma, value, goals, and factors impacting their mental health. Participants were also encouraged to complete the Managing Your Care module at some point, which discusses how to communicate and work with providers. If participants did not engage with the app at all within 7 days, they received one automatic notification as follows: “You haven’t logged into the Life Goals app in a while. Tap here to start working on a module.” If they left a module incomplete within the past 7 days and did not return to it, they received the following prompt: “You haven’t completed your module. Tap here to take you back to where you left off.” In all, participants received a total of 3 automated notifications for app inactivity.

**Figure 1:**
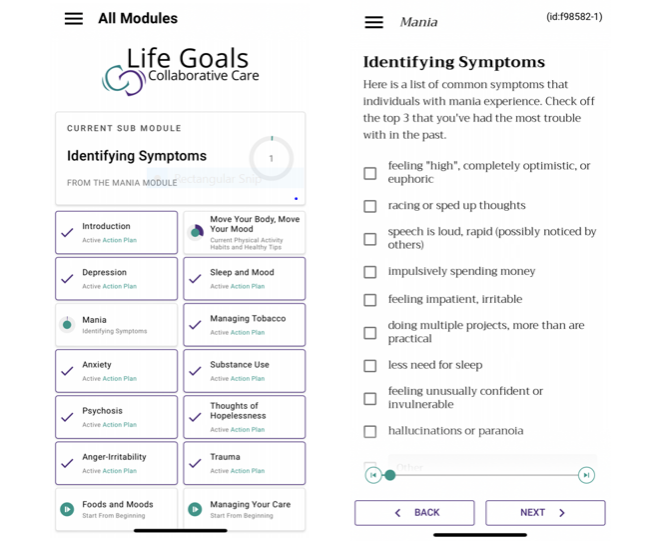
Life Goals app screenshots: landing page and Identifying Triggers (Depression module).

Following the mandatory introductory module, the other 13 Life Goals modules covered the following mental health and wellness topics: (1) Managing Your Care; (2) Depression; (3) Mania; (4) Anxiety; (5) Trauma; (6) Thoughts of Hopelessness; (7) Psychosis; (8) Anger/Irritability; (9) Substance Use; (10) Foods and Moods; (11) Move Your Body, Move Your Mood; (12) Managing Tobacco; and (13) Sleep and Mood. Each topic is customizable based on what the participant needs or wants to learn about. Participants were encouraged to complete at least 6 modules in all. See Figure 1 for example screenshots from the Life Goals app.

### Feasibility Assessment

Feasibility and patient engagement were assessed through data on the number of times the participants engaged with the Life Goals app and the number of minutes they spent using the Life Goals app during the 6-month study period. A post-study Questionnaire about the user’s experiences with the app was used to assess usability and acceptability and collected only at the 6-month interval. The survey contained 7 statements that participants rated their agreement with on a Likert scale, ranging from “Strongly Agree” to “Strongly Disagree.” With regard to the usability of the app, participants were asked to rate their agreement with the statements “The material was displayed adequately on my phone screen” and “I had problems accessing the app…because of technical difficulties.” Key statements relating to acceptability of the app as a self-management tool were, “The app improved my ability to manage my own health,” and “The app helped me make progress on my wellness goals” (see Table 1). Participants were also asked a single open-ended question, in response to which they could provide us with additional free-text feedback on their app use experience.

**Table 1:** Participant ratings on poststudy evaluation (n=25).

Evaluation question	Participants, n (%)		
	Strongly agree or agree	Neutral	Strongly disagree or disagree
1. The Life Goals app improved my ability to manage my own health.	9 (36)	10 (40)	6 (24)
2. The Life Goals app material was useful to me.	15 (60)	4 (16)	6 (24)
3. I enjoyed working through the Life Goals app material.	9 (36)	10 (40)	6 (24)
4. The Life Goals app helped me make progress on my wellness goals.	10 (40)	4 (16)	11 (44)
5. The Life Goals app was easy to use.	18 (72)	2 (8)	5 (20)
6. The material was displayed adequately on my phone screen.	22 (88)	1 (4)	2 (8)^a^
7. I had problems accessing the Life Goals app because of connectivity or other technical difficulties.	3 (12)	3 (12)	19 (76)

^a^Response: “Don’t know.”

### Outcome Assessment

Clinical outcomes were self-reported using validated measures of mood, health, and disability at 3 time points: baseline (study entry) and 3 and 6 months postinitiation of Life Goals app. A 6-month time span was chosen, as the original LGCC showed improvements in functioning after this time point [28]; however, in examining the feasibility of a stand-alone self-management app, we added a 3-month assessment period given that many individuals may lose interest after the first few months. A survey of the demographics (ie, age, education, race, and gender), current employment or living status, and insurance coverage was conducted only at study entry. Outcome measures assessed at all 3 waves included health-related quality of life, measured using the 12-item Short Form Health Survey (SF-12) [34]; self-reported health or disability status, measured using World Health Organization Disability Assessment Schedule 2.0 (WHODAS 2.0) [35]; alcohol use, measured using Alcohol Use Disorders Identification Test (AUDIT-C) [36]; drug use, measured using Drug Abuse Screening Test (DAST-10) [37]; and psychiatric symptoms, measured using the Patient Health Questionnaire–9 [38] for depression symptoms and the Internal State Scale [39] for mania or hypomania symptoms. Surveys took approximately 5-10 minutes to complete. All self-report measures were collected digitally using Research Electronic Data Capture (REDCap) tools hosted at University of Michigan [40].

### Compensation

Participants received monetary compensation for their participation in this study and to offset any data usage they may have accrued on their own mobile phones. Participants were offered US $1 for each day they used the app (defined as any interaction within the app), regardless of how many cell phone engagements they chose to use, or up to US $180 for 6 months. They also received US $10 for each survey completed, up to US $30. Maximum compensation for the 6-month study duration was US $210.

### Analyses

Univariate and bivariate statistics were used to examine all feasibility, usability, acceptability, and exploratory clinical outcomes. Feasibility was assessed by examining usage (defined as the number of instances the app was used and the duration while using the app and its modules) across the total 6-month study duration, from baseline to 3 months, and from 3 months to 6 months. For usability and acceptability estimates, frequencies of responses and open-ended qualitative responses were reviewed and summarized to determine common themes. For our exploratory clinical outcomes, paired *t*-tests were performed to evaluate over-time change. Spearman rho correlations were performed to determine associations between over-time change in outcome measures and app usage. 

## Results

### User Demographics

Of the 496 email invitations sent, 28 (5.6%) participants consented and enrolled in this study. The majority of participants were diagnosed with bipolar type I (n=21, 75%), with a smaller proportion diagnosed with bipolar type II (n=4, 14.3%) and bipolar, NOS (n=3, 10.7%). The average age of participants was 44.7 (range 24-72) years; 68% (19/28) identified as female; 75% (21/28) reported having a college degree; and 10.7% (3/28) identified as racial minorities (Multimedia Appendix 1).

### User Engagement

In this pilot study, app use waxed and waned over the course of the study. Of the 28 enrolled participants, 26 (93%) used the app at least once during the study period; 24 (86%) used the app during the first 3 months, and this number decreased to 18 (64%) during the next 3 months.

Over the full study duration, participants engaged with the app for a median of 25 times (IQR 13-65.75) and used the app for an average of 154 (IQR 39-194) minutes. However, this usage was not evenly distributed over time. During the first 3 months, participants engaged with the app for a median of 16.5 (IQR 12.25-41.25) times and for a median of 117 (IQR 22-132) minutes. During the next second 3 months, the median app use had increased to 23 (IQR 6-64.50) times, but the average time spent decreased to 66 minutes (IQR 22, 79) during the second period.

Among the subset of 18 participants who used the app at least once during the first and last 3-month periods, they also showed more app use within the first 3 months (median 11, IQR 7-28 instances) than in the last 3 months (median 8, IQR 5-35 instances), which was a statistically significant reduction (P=.005). This subset also used the app for longer, on average, in the first 3 months of the study (median 7, IQR 1-59 minutes per usage) compared to the last 3 months (median 3.4, IQR 1-31 minutes; P=.006).

### Module Completion

**Figure 2:**
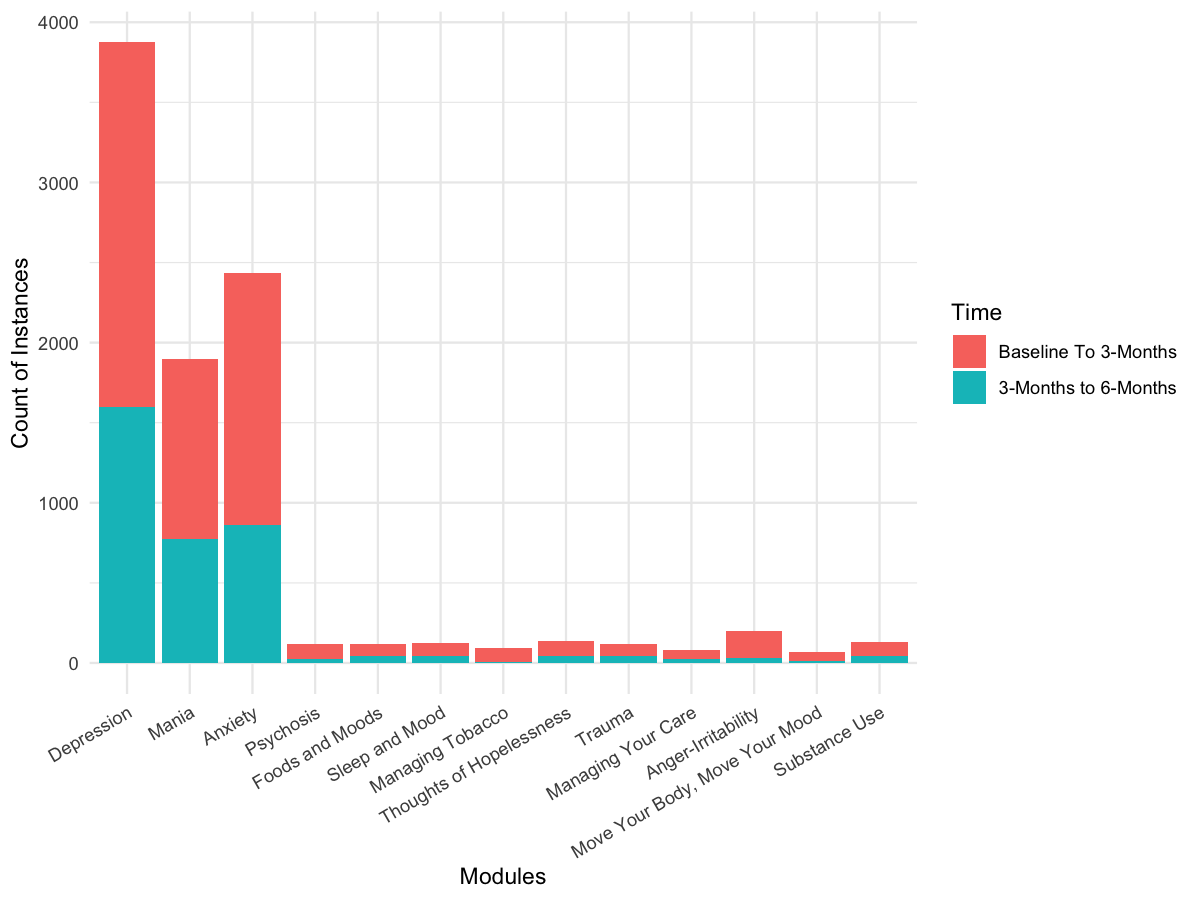
Number of times the Life Goals app was used at different time points.

Participants were required to complete the introduction module before attempting any other module. Furthermore, they were encouraged to use any or all modules, as deemed interesting, during the 6-month period. Of the 13 modules, excluding the mandatory introduction module, 3 (17%) participants used all 13 modules in the app. Over the course of the study, participants completed a number of modules (mean 8.2, SD 3.3; median 7; mode 5; range 4-13). All participants completed more than 3 Life Goals app modules, and 67% (12/18) of the participants completed 6 or more modules The most frequently assessed modules were the same in both study periods (ie, first 3 months and next 3 months); these included Depression, Anxiety, and Mania (Figure 2).

### Usability and Acceptability of Life Goals App

A total of 25 (89%) participants completed the poststudy survey about acceptability and usability (Table 1). Of these 25 participants, the majority found the material provided by the Life Goals app useful (n=15, 60%); they found the app easy to use (n=18, 72%); and they found the screen display adequate (n=22, 88%). Few participants reported problems accessing the Life Goals app because of connectivity or other technical difficulties (3/25, 12%). However, positive impacts of the app on health management were less widely endorsed: 10 (40%) users reported finding the app helpful in managing their health, and 9 (36%) reported finding it helpful in making progress on their wellness goals.

The open-ended question where participants could provide additional feedback on their app use experience included the following responses:

A lot of the information was obvious to me.

The app is well designed. I can see it being highly beneficial to someone learning how to manage their illness.

I’m pretty stable and didn't really find the app helpful…If I was experiencing symptoms, maybe more so.

I think it will help beginner sufferers more. My habits didn't change a lot, but Life Goals App really got me thinking differently.

### Outcome Assessment

Paired sample *t*-tests did not show significant changes in symptoms or functioning when comparing baseline assessments to 3-month follow-up assessments, or when comparing baseline assessments to 6-month follow-up assessments (Multimedia Appendix 2), likely due to the small sample size. Effect sizes were all small to negligible (Cohen *d* range –0.372 to 0.183). However, trends for improvements in physical health functioning, mental health functioning, and mania-related well-being were noted. There were no significant relationships between time spent using the app (minutes) or the number of instances using the app within the first 3 months or the last 3 months and changes in outcome measurements during the same periods, except for a positive correlation between change in mania symptoms and Life Goals app duration (see Multimedia Appendix 3).

## Discussion

### Principal Results

This pilot study examined the feasibility, usability, and acceptability of the Life Goals self-management app among individuals diagnosed with BD. We found that the median number of times that individuals used this app was 25, for a total of 154 minutes across 6 months. As with usage patterns of other apps, the app was used for a longer time during the first 3 months than the last 3 months of the study period. In a clinical setting, Life Goals participants are expected to complete 6 sessions, or 6 Life Goals modules; completion of 3 sessions is regarded as a clinically significant dose [29,41,42]. As such, all participants completed at least 3 Life Goal app modules, and the majority completed at least 6 Life Goals modules, indicating all received a clinically significant dose comparable to Life Goals in a clinical setting. Furthermore, the number of modules completed (mean 8.2, median 7) indicated reasonable use of the modules. Not counting the introduction module, which was mandatory, the most frequently accessed modules in our sample were Depression, followed by Anxiety and Mania, suggesting that these are areas that appealed to the sample the most or directly addressed self-management needs.

For usability and acceptability, frequencies of responses from participants indicated high usability and satisfaction with the user interface. The majority of participants experienced no difficulties using the app and felt that the material was displayed adequately; however, the results showed low success in the app encouraging self-management of their own health. Only a minority of the participants felt that the app helped them to make progress on their wellness goals and improved their ability to manage their own health. Similarly, when symptoms, health, and functioning were rated over the course of the study using self-report surveys, we found no significant improvements in ratings. However, there were notable trends for mania and mental and physical health functioning over the first 3 months, using this small sample that were pointing in the direction of improved scores. Written responses from participants centered around themes related to liking the app, but the content or self-management pieces were not novel to them, and a few suggested that they felt that the app would be more beneficial to individuals who were newly diagnosed. These findings suggest that targeting the app to those who have received less psychoeducation about BD or were earlier in their illness course may find greater benefit. Future work to understand what individuals with BD look for in similar apps is needed. Only 6% of all those who offered to participate in this study agreed to use the app, suggesting that there may be specific factors that influence the uptake of this type of app. Our future work aims to explore both dissemination via direct-to-consumer and clinically integrated pathways that would address the question about uptake and engagement with self-management apps, such as Life Goals. Recent work suggesting design considerations for development, engagement, and evaluation [43] in apps for BD will be helpful to refine the development and dissemination of this work.

### Limitations

Given our small sample size, our group of participants with BD may not be reflective of the broader, heterogeneous nature of BD. Furthermore, it is possible that a larger sample would have sufficient power to detect changes in clinical outcomes. We did not include a control group, so it was not possible to determine whether clinical changes were related to use of the Life Goals app. Our study design likely resulted in the tendency to include individuals who are more technology oriented and motivated to participate in research, as well as those who are more willing to have an almost daily engagement with the Life Goals app. For example, our sample was already actively engaged in a longitudinal study of BD, so these participants are likely more willing to engage in other research studies and participate in daily activities than are other individuals who are not volunteering their time. Future studies will need to evaluate the use of the Life Goals app in a more rigorous or controlled manner and within a more generalizable population to examine whether use statistics were related to incentivizing participants or whether symptom improvement trends persist over time. Lastly, we compensated our participant’s time in this study, which may have increased their interest to participate or their motivation to engage with the Life Goals app.

### Conclusions

Overall, these results indicate that the Life Goals app, a smartphone self-management app developed using an evidence-based intervention based on the chronic collaborative care model, is feasible and acceptable for individuals with BD. Individuals using the Life Goals app may use it more frequently at first and may have the greatest interest in mood-related modules rather than other wellness modules. Wider dissemination of the app to individuals in different stages of recovery or earlier in the illness is needed. This app shows potential as an mHealth technology based on evidence-based treatments that can help bridge the gap in access to care and reducing burden on providers. It can also offer greater flexibility in terms of treatment options that accommodates varying levels of user engagement and commitment.

## Appendix

Multimedia Appendix 1 Demographic characteristics of the study participants (N=28).

Multimedia Appendix 2 Self-report clinical outcome data with completion rate, mean score, and SD at each time point.

Multimedia Appendix 3 Correlations between Life Goals app use and outcome measures for time period 1 (ie, baseline to 3-month follow-up) and time period 2 (ie, 3-month to 6-month follow-up).

## Abbreviations

Audit-C: Alcohol Use Disorders Identification Test
BD: bipolar disorder
DAST-10: Drug Abuse Screening Test
DSM-IV: Diagnostic Statistic Manual version IV
LGCC: Life Goals Collaborative Care
mHealth: mobile health
NOS: not otherwise specified
REDCap: Research Electronic Data Capture
SF-12: 12-item Short Form Health Survey
WHO-DAS: World Health Organization Disability Assessment Schedule 2.0
