# Supplementary material for: The Life Goals Self-Management Mobile App for Bipolar Disorder: Consumer Feasibility, Usability, and Acceptability Study
Source: JMIR Form Res. 2021 Dec 13;5(12):e32450. doi: 10.2196/32450 (PMC8713087; doi:10.2196/32450)
Supplement: Multimedia Appendix 1 [file formative_v5i12e32450_app1.docx]

| Demographic characteristics of the study participants (N=28). | | | |
| --- | --- | --- | --- |
| Age (M/SD) | 44.71 (11.251) |  |  |
| Gender (n/% female) | n=19; 67.90% |  |  |
| Level of education | High school/GED – n=3; 10.70%  Some College – n=4; 14.30%  Technical training – n=1; 3.60%  Bachelor’s degree – n=11; 39.30%  Graduate Degree – n=9; 32.1% |  |  |
| Race (n/% majority/minority) | White: n =25 (89.30%); Black: n=2 (7.1%); Asian = 1 (3.60%) |  |  |
| Engaged in Community mental Health (%) | n = 9 (32.10%) |  |  |
| Type BD (# BDI/#BDII/#BDNOS) | 21 BDI (75.00%); 4 BDII (14.30%); 3 BD NOS (10.70%) |  |  |
| HRDS (M/SD) (at study entry) 17-item | 8.24 (8.23) |  |  |
| YMRS (M/SD) (at study entry) | 4.95 (6.16) |  |  |
| Phone given (Yes) | 32.1% |  |  |
| *Clinical features for the BD group* |  |  |  |
| Length with BD illness (M/SD) | 26.74 years (12.02) |  |  |
| Rapid Cycling (% yes) | n=3; 10.70% |  |  |
| Psychosis history (% yes) | n = 15; 53.60% |  |  |
| Comorbidities | 3.12 (1.51) |  |  |

BD=Bipolar Disorder; I=Type I, II=Type II, NOS=Not Otherwise Specified
