# Supplementary material for: The Life Goals Self-Management Mobile App for Bipolar Disorder: Consumer Feasibility, Usability, and Acceptability Study
Source: JMIR Form Res. 2021 Dec 13;5(12):e32450. doi: 10.2196/32450 (PMC8713087; doi:10.2196/32450)
Supplement: Multimedia Appendix 2 [file formative_v5i12e32450_app2.docx]

| Self-report clinical outcome data with completion rate, mean score, and SD at each time point. | | | | | |
| --- | --- | --- | --- | --- | --- |
| *Outcome Measures (M/SD)* | Baseline (n=27) | 3 month (n=25) | 6 month (n=25)^a^ | Baseline to 3 month  *t* test (*df*), *P* value | Baseline to 6 month  *t* test (*df*), *P* value |
| SF-12 PCS | 43.80 (11.21) | 43.41 (12.59) | 42.27 (12.51) | -0.380, .707 | 1.545, .136 |
| SF-12 MCS | 39.78 (12.49) | 37.69 (14.46) | 38.65 (12.63) | 1.679, .107 | 0.818, .422 |
| PHQ-9 | 9.19 (6.99) | 8.52 (7.27) | 9.12 (5.80) | 0.597, .557 | -0.200, .843 |
| ISS-a score | 116.67 (144.75) | 92.80 (116.64) | 125.20 (128.33) | 1.353, .189 | -0.042, .967 |
| ISS-wb score | 157.78 (74.39) | 135.60 (80.32) | 140.00 (72.57) | 1.549, .135 | 1.771, 0.90 |
| WHO-DAS | 26.62 (21.3) | 26.33 (22.27) | 29.00 (20.04) | 0.531, .600 | -0.847, .406 |
| Audit-C | 1.74 (2.94) | 2.08 (3.62) | 2.00 (2.93) | -0.459, .651 | -1.634, .116 |
| DAST-10 | 1.00 (1.84) | 0.64 (1.38) | 0.60 (1.12) | 0.911, .372 | 0.000, 1.00 |

SF-12 PCS=12-item Short Form Health Survey Physical Health Component Score; SF-12 MCS=12-item Short Form Health Survey Mental Health Component Score; PHQ-9= Patient Health Questionnaire – 9; ISS= The Internal State Scale (a=activation score; wb=well-being score); WHO-DAS=World Health Organization Disability Assessment Schedule 2.0; Audit-C=Alcohol Use Disorders Identification Test (^a^: n=19); DAST-10= Drug Abuse Screening Test
