# Supplementary material for: The Life Goals Self-Management Mobile App for Bipolar Disorder: Consumer Feasibility, Usability, and Acceptability Study
Source: JMIR Form Res. 2021 Dec 13;5(12):e32450. doi: 10.2196/32450 (PMC8713087; doi:10.2196/32450)
Supplement: Multimedia Appendix 3 [file formative_v5i12e32450_app3.docx]

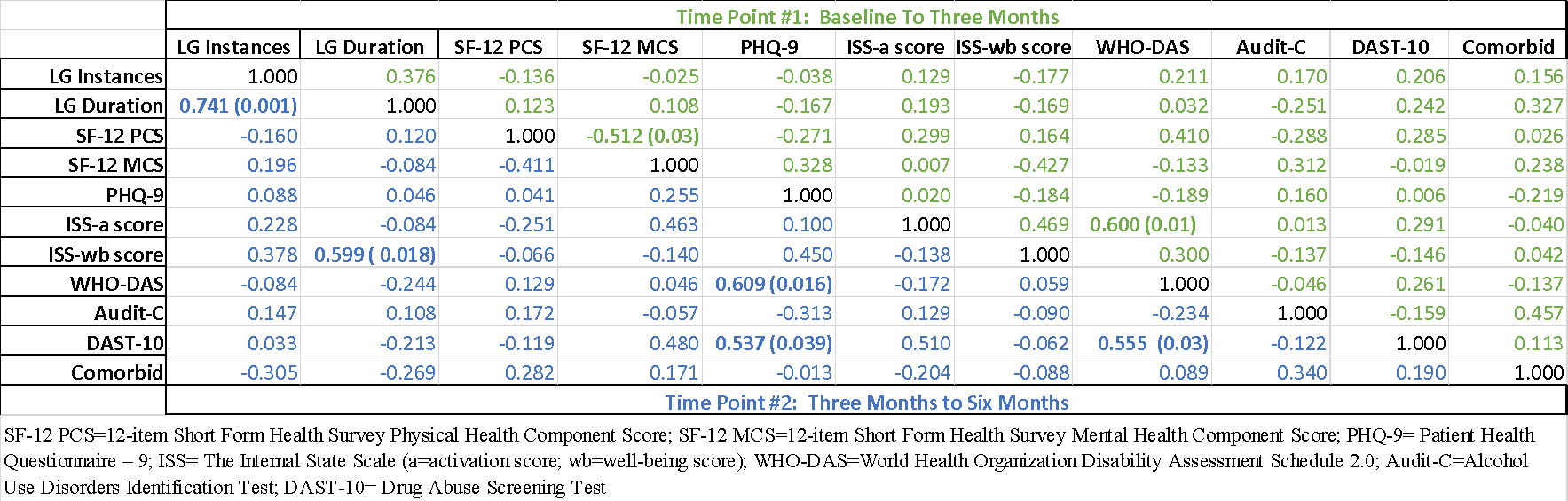


Correlations between Life Goals app use and outcome measures for time period 1 (ie, baseline to 3-month follow-up) and time period 2 (ie, 3-month to 6-month follow-up)
